# Resting sympathetic baroreflex sensitivity in subjects with low and high tolerance to central hypovolemia induced by lower body negative pressure

**DOI:** 10.3389/fphys.2014.00241

**Published:** 2014-06-30

**Authors:** Carmen Hinojosa-Laborde, Kathy L. Ryan, Caroline A. Rickards, Victor A. Convertino

**Affiliations:** ^1^U.S. Army Institute of Surgical ResearchFort Sam Houston, TX, USA; ^2^University of North Texas Health Science CenterFort Worth, TX, USA

**Keywords:** hemorrhage, orthostatic intolerance, autonomic reflex

## Abstract

Central hypovolemia elicited by orthostasis or hemorrhage triggers sympathetically-mediated baroreflex responses to maintain organ perfusion; these reflexes are less sensitive in patients with orthostatic intolerance, and during conditions of severe blood loss, may result in cardiovascular collapse (decompensatory or circulatory shock). The ability to tolerate central hypovolemia is variable and physiological factors contributing to tolerance are emerging. We tested the hypothesis that resting muscle sympathetic nerve activity (MSNA) and sympathetic baroreflex sensitivity (BRS) are attenuated in male and female subjects who have low tolerance (LT) to central hypovolemia induced by lower body negative pressure (LBNP). MSNA and diastolic arterial pressure (DAP) were recorded in 47 human subjects who subsequently underwent LBNP to tolerance (onset of presyncopal symptoms). LT subjects experienced presyncopal symptoms prior to completing LBNP of −60 mm Hg, and subjects with high tolerance (HT) experienced presyncopal symptoms after completing LBNP of −60 mm Hg. Contrary to our hypothesis, resting MSNA burst incidence was not different between LT and HT subjects, and was not related to time to presyncope. BRS was assessed as the slope of the relationship between spontaneous fluctuations in DAP and MSNA during 5 min of supine rest. MSNA burst incidence/DAP correlations were greater than or equal to 0.5 in 37 subjects (LT: *n* = 9; HT: *n* = 28), and BRS was not different between LT and HT (−1.8 ± 0.3 vs. −2.2 ± 0.2 bursts·(100 beats)^−1^ ·mm Hg^−1^, *p* = 0.29). We conclude that tolerance to central hypovolemia is not related to either resting MSNA or sympathetic BRS.

## Introduction

Central hypovolemia occurs during orthostasis (upright posture) as blood pools in the legs due to gravity, and also during hemorrhage as blood volume decreases. Low tolerance to central hypovolemia has critical implications during both circumstances. Patients with orthostatic intolerance experience symptoms ranging from lightheadedness and postural instability to loss of consciousness upon standing (Stewart, 2013). Trauma patients with low tolerance to hypovolemia will reach the point of cardiovascular collapse (hemodynamic instability resulting from the inability of cardiovascular mechanisms to compensate for blood loss) in a shorter amount of time for similar blood loss, and thus may require life-saving interventions sooner than patients with high tolerance. Using lower body negative pressure (LBNP) as an experimental model to induce central hypovolemia, we have shown that approximately 1 out of 3 human subjects has low tolerance to this stress (Convertino and Sather, 2000a,b; Rickards et al., 2011). If extended to the clinical setting, these results suggest that as many as one-third of hemorrhaging trauma patients are at risk for early and/or rapid development of hypotension and overt circulatory shock. Identifying physiological and/or physical factors which influence tolerance may help elucidate the mechanism, and lead to potential identification of individuals with low tolerance to central hypovolemia prior to the onset of symptoms.

Tolerance to central hypovolemia is associated with maintenance of adequate perfusion pressure (i.e., arterial blood pressure) to vital organs. Therefore, we hypothesize that the baseline sensitivity of the autonomic baroreflex responsible for the control of cardiac output and peripheral vascular resistance would be less sensitive in individuals who display low tolerance to central hypovolemia. Indeed, numerous investigations have verified that predominantly parasympathetically-mediaed cardiac baroreflex responses at rest are associated with orthostatic tolerance (Convertino et al., 1990; Engelke et al., 1992, 1994, 1995, 1996; Convertino, 2002; Schafer et al., 2013). However, the relationship between resting sympathetically-mediated reflex responsiveness and orthostatic tolerance is less studied.

A mechanism important for compensation to hypovolemia is activation of the sympathetic nervous system which produces peripheral vasoconstriction (Rowell, 1986). The progressive increase in MSNA, and subsequently, peripheral vascular resistance during various levels of hypovolemia is well documented (Johnson et al., 1974; Sundlof and Wallin, 1978; Rowell, 1986; Pawelczyk and Raven, 1989; Khan et al., 2002; Kimmerly and Shoemaker, 2002; Convertino et al., 2004; Ichinose et al., 2004; Cooke et al., 2009) In LBNP studies, subjects with low tolerance (LT) and high tolerance (HT) have similar resting vascular resistance (Sather et al., 1986; Convertino and Sather, 2000b; Convertino et al., 2012), but LT subjects have a diminished vasoconstrictor response between baseline and maximum LBNP, i.e., a diminished vasoconstrictor reserve (Engelke et al., 1996; Convertino and Sather, 2000b; Fu et al., 2004; Convertino et al., 2012; Cote et al., 2012). This diminished vasoconstrictor reserve has also been observed in patients with orthostatic intolerance (Harrison, 1986; Waters et al., 2002). As with vascular resistance, resting muscle sympathetic nerve activity (MSNA) is similar in LT and HT subjects, but the increase in MSNA during low levels of LBNP, and at the time of presyncope is less in LT subjects (Wasmund et al., 2003; Convertino et al., 2012; Fu et al., 2012).

The regulation of vascular resistance by MSNA is primarily accomplished via the sympathetic baroreflex. The sensitivity of the sympathetic baroreflex can be assessed by linear regression of the spontaneous fluctuations in MSNA and arterial pressure (Kienbaum et al., 2001). There are no previous studies which correlate resting MSNA and MSNA BRS with absolute tolerance time in subjects exposed to LBNP to presyncope. We hypothesize that the diminished capacity of LT subjects to increase MSNA and vascular resistance during LBNP is due to a reduced resting sympathetic baroreflex gain, and thus can distinguish between LT and HT subjects prior to the onset of LBNP.

## Methods

### LBNP protocol

The protocol was approved by the Institutional Review Board of the Brooke Army Medical Center, Fort Sam Houston, TX. All studies were conducted at the U.S. Army Institute of Surgical Research, Fort Sam Houston, TX. Normotensive, non-smoking healthy human volunteers (*N* = 47; 35 males/12 females) participated in this study after evaluation of their medical history and physical examination by a physician to ensure the absence of previous and current medical conditions that would exempt them as participants. Subjects were instrumented for standard lead II electrocardiogram to record heart rate (HR), and a finger photoplethsmography cuff (Finometer®) to record beat-by-beat finger systolic, mean and diastolic arterial pressure (SAP, MAP, DAP). MSNA was measured via microneurography of the peroneal nerve (Hagbarth and Vallbo, 1968). Nerve signals were band-pass filtered (100–2000 Hz) and integrated (time constant, 0.1 s) to obtain mean voltage neurograms.

**Figure 1:**
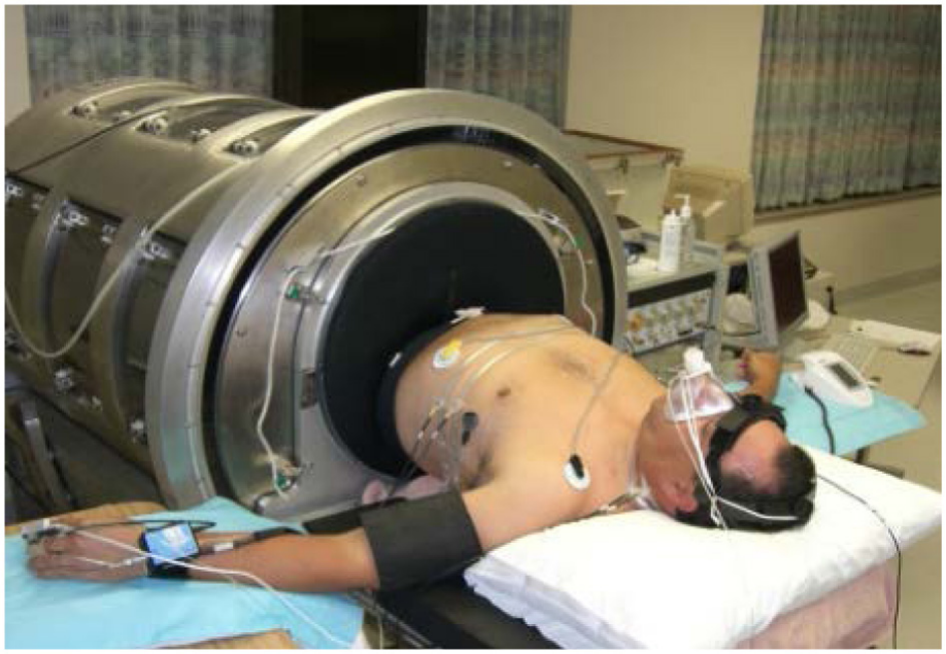
**Photograph of subject in the LBNP chamber**.

To simulate hemorrhage in conscious humans, central hypovolemia was induced by application of LBNP (Figure 1). Subjects were positioned supine within an airtight chamber that was sealed at the level of the iliac crest by a neoprene skirt, and allowed to acclimate to this position for 10–20 min prior to initiating the study. The LBNP protocol consisted of a 5-min control period (baseline) followed by stepwise chamber decompression of −15, −30, −45, −60, −70, −80, −90, and −100 mm Hg. LBNP was increased every 5 min until the onset of cardiovascular collapse. Cardiovascular collapse was defined by one or a combination of the following criteria: (a) sudden bradycardia, (b) a precipitous fall in systolic pressure greater than 15 mm Hg; (c) progressive fall of systolic pressure below 80 mm Hg; and, (d) voluntary termination due to the onset of presyncopal symptoms such as sweating, nausea, dizziness, vision alterations, or general discomfort. Time to presyncope (TTP) was calculated as the elapsed time (seconds) from the start of baseline to termination of LBNP. Based on the above-mentioned hemodynamic criteria for presyncope or subject reporting of presycopal symptoms, all 47 subjects reached presyncope.

### Data analysis

**Figure 2:**
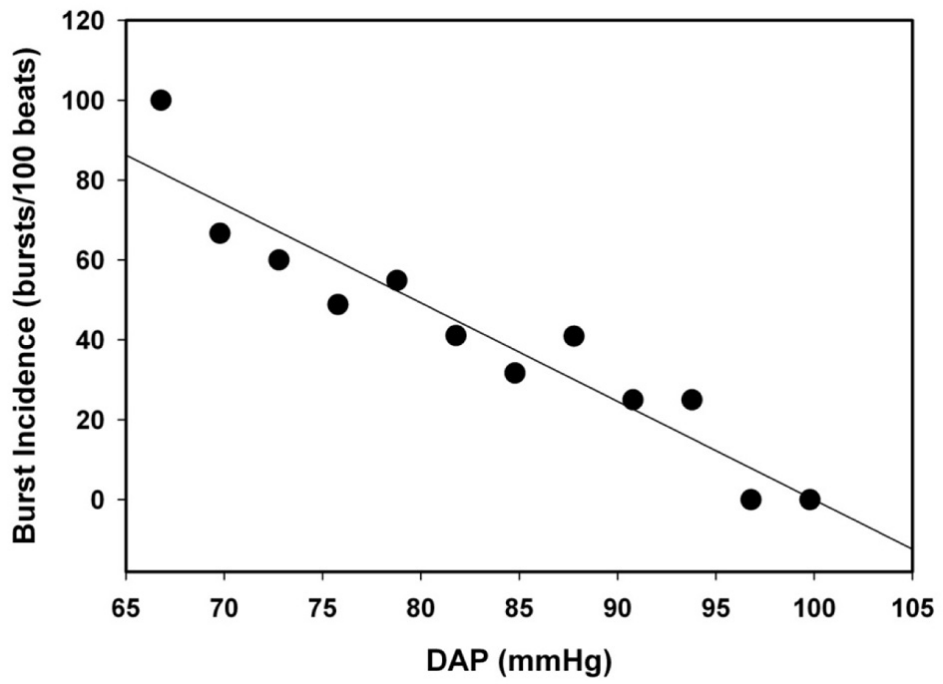
**Sample relationship between DAP and MSNA burst incidence (*r* = 0.88)**.

Subjects were divided into two groups based on TTP. Subjects who were able to complete −60 mm Hg LBNP with TTP ≥ 1500 s were classified as HT, and subjects who were not able to complete −60 mm Hg LBNP with TTP < 1500 s were classified as LT. Data were sampled at 500 Hz and digitally acquired using WINDAQ (Dataq Instruments, Akron, OH), and all analysis preformed with Win CPRS (Absolute Aliens, Turku, Finland). MSNA was quantified by burst incidence (number of bursts per 100 heart beats). Correlation between resting MSNA and LBNP tolerance (TTP) was determined by linear regression analysis. Spontaneous sympathetic baroreflex sensitivity (BRS) was calculated as the slope of the linear regression between spontaneous fluctuations in DAP (3 mm Hg bins) and MSNA during a 5 min baseline period using WinCPRS (Kienbaum et al., 2001). Linear regression analysis was performed on all subjects for MSNA burst incidence/DAP; however, only subjects with correlation coefficients greater than or equal to 0.5 were evaluated for BRS (Ogoh et al., 2009). Differences in proportions of LT and HT in which BRS was calculated were determined by *z*-test for proportions. Figure 2 shows the linear regression analysis on DAP and MSNA burst incidence in one subject with a slope (BRS) of 2.5 (bursts/100 beats/mm Hg) and a correlation coefficient of 0.88. Statistical differences in BRS between LT and HT were determined by *t*-test analysis.

## Results

### Hemodynamic and MSNA values in LT and HT subjects

**Figure 3:**
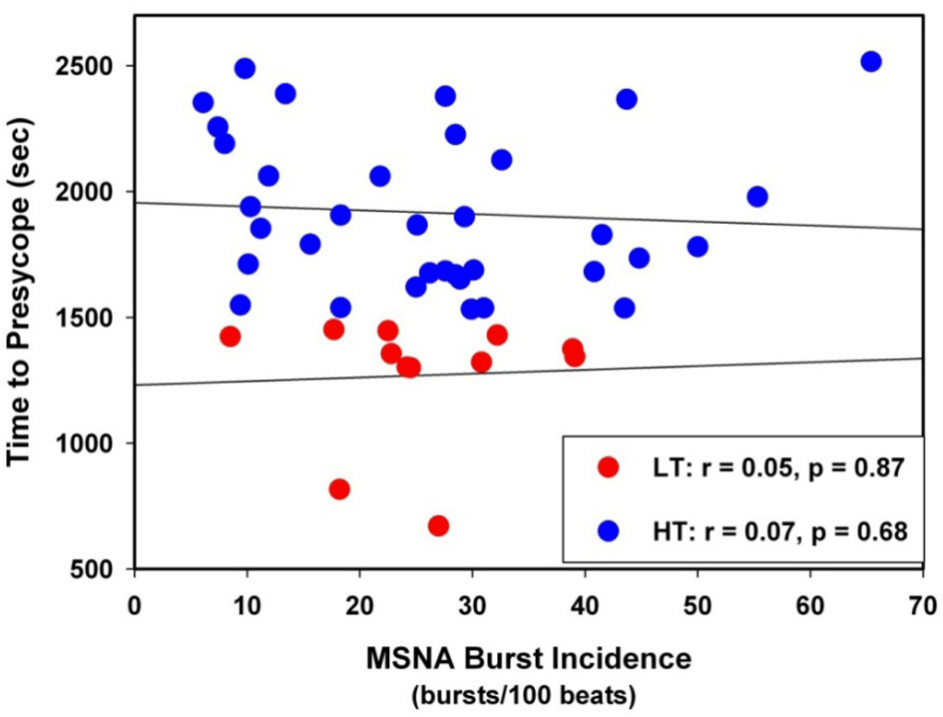
**Linear regressions between MSNA burst incidence and time to presyncope in low tolerant (LT) and high tolerant (HT) subjects**.

By design, the TTP was lower in LT (*n* = 12) compared with HT (*n* = 35) subjects (Table 1). Resting values of HR, SAP, MAP, DAP, and MSNA burst incidence were statistically indistinguishable (*P* ≥ 0.28) between LT and HT subjects. Resting values of MSNA burst incidence in LT and HT subjects were not related to TTP (Figure 3).

**Table 1:** **Hemodynamic and MSNA variables at baseline**.

**Variable**	**LT**	**HT**	***P*-value**
N	12	35	
Male/Female	9/3	26/9	
Time to presyncope	1269 ± 76	1916 ± 52	*P* < 0.001
HR (beats/min)	66 ± 3	63 ± 1	*P* = 0.28
SAP (mm Hg)	129 ± 2	131 ± 2	*P* = 0.59
MAP (mm Hg)	96 ± 2	97 ± 1	*P* = 0.82
DAP (mm Hg)	75 ± 2	76 ± 1	*P* = 0.62
MSNA burst incidence (bursts/100 heart beats)	25.5 ± 2.7	26.5 ± 2.5	*P* = 0.84
MSNA burst incidence/DAP slope (bursts·100 beats^−1^ ·mm Hg^−1^)	−1.8 ± 0.3 (*n* = 9)	−2.2 ± 0.2 (*n* = 28)	*P* = 0.29

Data are shown as mean ± s.e.m. for time to presyncope, HR, heart rate; SAP, systolic arterial pressure; MAP, mean arterial pressure; DAP, diastolic arterial pressure; MSNA, muscle sympathetic nerve activity burst incidence, and the slope of the relationship between MSNA burst incidence and DAP in subjects with low tolerance (LT) and high tolerance (HT) to central hypovolemia. P-values for t-test comparisons between LT and HT are shown for each variable.

### MSNA baroreflex sensitivity in LT and HT

**Figure 4:**
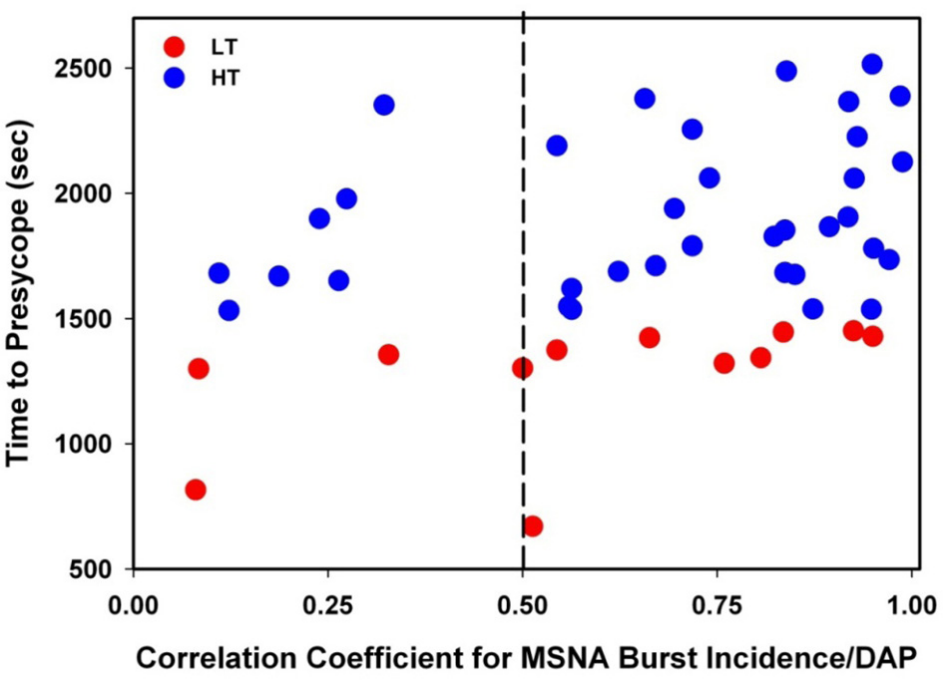
**Correlation coefficients for MSNA burst incidence/DAP slopes plotted against time to presyncope in low tolerant (LT) and high tolerant (HT) subjects**. Only subjects with correlation coefficients ≥0.5 were evaluated for MSNA baroreflex sensitivity by burst incidence (75% of LT; 80% of HT; *p* = 0.83).

**Figure 5:**
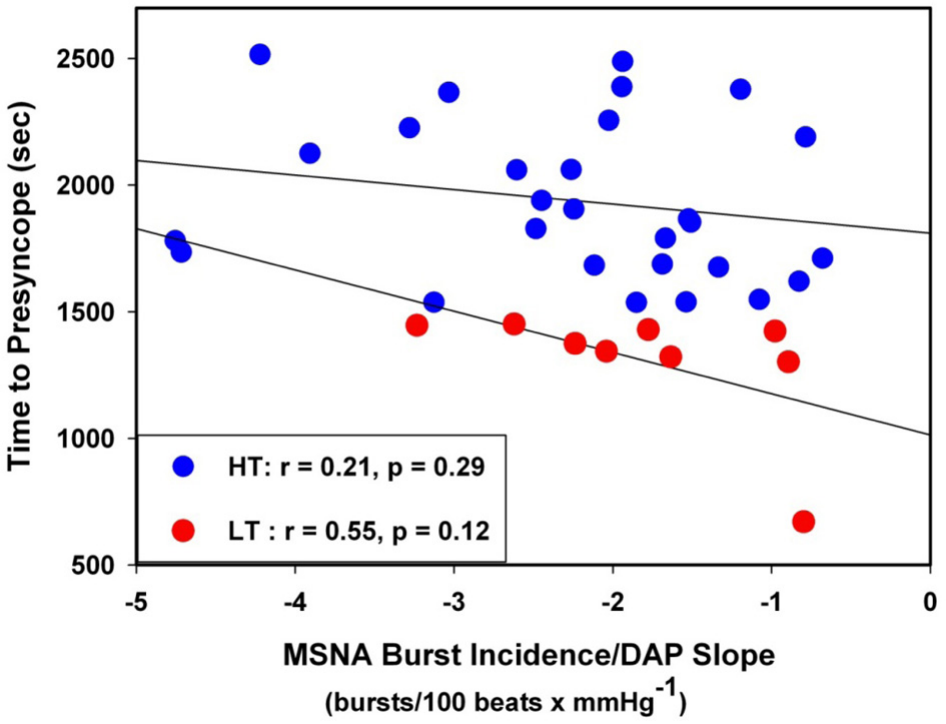
**Linear regressions between MSNA burst incidence/DAP slope and time to presyncope in low tolerant (LT) and high tolerant (HT) subjects**.

The individual correlation coefficients from the linear regression analyses for MSNA burst incidence/DAP compared to TTP in all subjects are shown in Figure 4. Only subjects with correlation coefficients greater than or equal to 0.5 were evaluated for MSNA BRS by burst incidence (75% of LT, *n* = 9; 80% of HT, *n* = 28; *p* = 0.83). MSNA BRS at baseline, assessed as the slope of MSNA burst incidence/DAP was not statistically different between LT and HT subjects (Table 1). MSNA BRS and TTP were weakly correlated in both HT and LT subjects (Figure 5).

## Discussion

This study assessed the correlation between resting MSNA and MSNA BRS with absolute tolerance time to acute central hypovolemia. Tolerance to acute hypovolemia is an intrinsic characteristic of an individual's complex physiological response to changes in central blood volume. The ability to identify or predict an individual's tolerance to hypovolemia PRIOR to any changes in blood volume status, could significantly alter how the individual is treated in the event of orthostatic instability or hemorrhage, potentially improving their clinical outcome. Based on previous studies showing that LT subjects have a diminished vasoconstrictor response and an attenuated increase in MSNA during simulated hypovolemia (Wasmund et al., 2003; Convertino et al., 2012; Fu et al., 2012), we hypothesized that baroreflex control of MSNA prior to LBNP would also be less in LT than HT subjects. To test this hypothesis, we assessed the relationship between spontaneous changes in MSNA in response to changes in diastolic blood pressure as an index of arterial baroreflex function, and tolerance to central hypovolemia. Contrary to our hypothesis, our assessment of sympathetic BRS during baseline in LT and HT subjects indicated that baroreflex control of MSNA was similar in both groups. In addition, resting levels of MSNA and MSNA BRS were unrelated to LBNP tolerance time. Considering our previous studies showing that cardiac baroreflex sensitivity is attenuated in LT subjects (Convertino et al., 1990, 1991; Convertino, 1991, 2002; Engelke et al., 1992, 1994, 1995, 1996; Schafer et al., 2013) the results of the current study support the observation that there is no correlation between cardiac and sympathetic arterial baroreflex sensitivities (Dutoit et al., 2010).

The hemodynamic and autonomic responses to orthostatically-induced central hypovolemia have been extensively studied. LBNP induces a redistribution of blood volume from the torso to the legs resulting in reductions in stroke volume, central venous pressure and pulse pressure (Cooke et al., 2004). Cardiopulmonary and arterial baroreceptors sense the change in central blood volume and respond by activating the sympathetic nervous system to increase vascular resistance and heart rate (Ichinose et al., 2006). Subsequently, blood pressure and cardiac output remain relatively stable until the most severe levels of central hypovolemia are attained. At this point of impending cardiovascular collapse, an abrupt decrease in blood pressure and vascular resistance is observed and presyncopal symptoms are typically experienced with concomitant decrease in cerebral blood flow (Cooke et al., 2004). Within this context, it is reasonable to hypothesize that individuals who have the greatest reserve to compensate (i.e., HT subjects) to a hypovolemic challenge would also display a greater baseline BRS of the sympathetic control for increasing vascular resistance. Against expectations, we did not find this to be the case.

A consistent observation in studies of experimentally induced hypovolemia is great variability in individual tolerance to this hemodynamic stressor. In addition to a blunted cardiac BRS response at baseline and during orthostasis (Convertino et al., 1990; Engelke et al., 1992, 1995; Convertino, 2002; Schafer et al., 2013), studies have revealed that LT subjects have a reduced capacity to increase vascular resistance and MSNA during LBNP (Convertino, 2002; Convertino et al., 2012; Cote et al., 2012; Fu et al., 2012). This observation led us to hypothesize that baseline sympathetic BRS may also be reduced in LT subjects, representing a smaller reserve to increase peripheral vascular resistance. We are unaware of any previous LBNP studies designed to correlate resting MSNA and MSNA BRS with absolute tolerance time in an LBNP study in which all subjects experienced presyncope. Convertino and co-workers (Convertino et al., 2012) reported no differences in sympathetic activity at baseline in HT and LT subjects, but did not measure sympathetically-mediated BRS. Ichinose et al. (Ichinose et al., 2006) reported MSNA and MSNA BRS at rest and during LBNP in a small number of syncopal and non-syncopal subjects exposed of a maximum of −60 mm Hg LBNP. While they demonstrated the dynamic nature of MSNA BRS during LBNP, they found no differences in resting MSNA BRS (Ichinose et al., 2006). Wasmund et al. (2003) conducted a study of tolerance to LBNP based on a positive or negative presyncopal response to 30 min of −60 mm Hg LBNP. They assessed BRS by nitroprusside and phenylephrine administration, and found that BRS at rest was not different between positive and negative responders. Similarly, using a tilt test to induce central hypovolemia, Fu et al. (2012) demonstrated that spontaneous sympathetic BRS at rest was not different between presyncopal and non-presyncopal subjects. Despite the difference in experimental design and method of calculating BRS, our data support the findings of Wasmund et al. (2003), Ichinose et al. (2006), Fu et al. (2012), and further demonstrate that the absolute time to presyncope is not correlated with resting MSNA or sympathetic BRS.

The assessment of MSNA BRS from spontaneous fluctuations in DAP and MSNA at baseline is limited by the relatively small stimulus range of DAP (approximately 15 mm Hg). As such, this comparison of baroreflex control of MSNA between HT and LT subjects is based on the assumption that our measurements included the linear portion around the operating point of the baroreflex function curve, rather than closer to threshold or saturation of the curve. Although this is a reasonable assumption during baseline (Figure 2), we have no data to either support or refute this assumption during LBNP. In summary, unlike vagally-mediated cardiac BRS, baroreflex control of MSNA at baseline does not differ between LT and HT individuals. This finding is important because it supports the notion that the greater vasoconstrictor reserve reported in HT individuals is solely dictated by their capacity to elicit a maximal sympathetic response during stress independent of baroreflex control.

## Disclaimer

The opinions or assertions contained herein are the private views of the authors, and are not to be construed as official or as reflecting the views of the Department of the Army or the Department of Defense.

### Conflict of interest statement

The authors declare that the research was conducted in the absence of any commercial or financial relationships that could be construed as a potential conflict of interest.
